# An Isometric and Functionally Based 4-Stage Progressive Loading Program in Achilles Tendinopathy: A 12-Month Pilot Study

**DOI:** 10.1155/2022/6268590

**Published:** 2022-05-24

**Authors:** Thøger Persson Krogh, Thomas Theis Jensen, Merete Nørgaard Madsen, Ulrich Fredberg

**Affiliations:** ^1^Diagnostic Centre, University Research Clinic for Innovative Patient Pathways, Silkeborg Regional Hospital, Silkeborg, Denmark; ^2^Department of Sports Medicine, Elective Surgery Centre, Silkeborg Regional Hospital, Silkeborg, Denmark; ^3^Center for Sports Medicine, Regional Hospital of Northern Denmark, Hjørring, Denmark; ^4^Interdisciplinary Research Unit, Elective Surgery Centre, Silkeborg Regional Hospital, Silkeborg, Denmark; ^5^Research Unit of Rheumatology, Department of Clinical Research, University of Southern Denmark, Odense University Hospital, Odense, Denmark; ^6^Institute of Sports Medicine Copenhagen, Bispebjerg Hospital, Copenhagen, Denmark

## Abstract

**Background:**

Achilles tendinopathy (AT) is a common musculoskeletal disorder, and its management remains challenging. *Hypothesis/Purpose*. By conducting a pilot study, we aimed to assess the feasibility, safety, and clinical improvement of a new home-based 4-stage rehabilitation program with progressive loading including isometric exercises on a small scale prior to setting up a randomized controlled trial.

**Methods:**

Ten recreational athletes with chronic midportion AT were included. The primary outcome was change in VISA-A score after 1, 2, 3, 6, and 12 months. Secondary outcomes included tenderness on palpation of the tendon and ultrasonographic changes after 6 months.

**Results:**

Average VISA-A improvements of 26.9 points (*P*=0.004) and 35.4 points (*P*=0.006) were observed at 6- and 12-month follow-up, respectively. Tenderness on palpation of the tendon (0–10) was reduced from 5.5 to 2.5 (*P* < 0.001). Color Doppler ultrasound activity (0–4) was reduced by 50%, from an average of grade 2 to grade 1 (*P*=0.023). The hypoechoic cross-sectional area of the Achilles tendon was reduced from an average of 29.1% to 8.5% (*P*=0.001). Tendon thickness showed no statistically significant change (*P*=0.415).

**Conclusion:**

Following the 4-stage rehabilitation program for AT based on isometric training and progressive loading, we observed improvement in both VISA-A score and ultrasonography in a group of athletes who had previously failed to benefit from standard AT rehabilitation. The study was feasible in terms of high adherence to the program and with no observed safety issues. The results of this pilot study support a further assessment of this specific approach for rehabilitation in a future randomized controlled trial.

## 1. Introduction

Chronic midportion Achilles tendinopathy (AT) is a common musculo-tendinous disorder that affects both competitive and recreational athletes, but it can also involve the non-athletic population [1, 2]. In a general practice cohort study, 35% of AT cases were related to sporting activities. The incidence rate was 1.85 per 1,000 registered patients, the mean age was 43.4 years, and 52.3% were females [3]. In certain sports such as running, high incidence of AT can be observed, e.g. the annual incidence in top-level runners is 9% [4]. AT is the preferred term for persistent Achilles tendon pain and loss of function related to mechanical loading [5]. Overload injury is the most widely accepted theory behind the development of AT and typically follows minor and often unrecognized trauma (microtrauma) [6].

As is the case for most tendinopathies, AT can be a difficult disorder to treat, with a combination of off-loading and rehabilitation being the most commonly recommended interventions [7–12]. Previous studies have shown that eccentric training and heavy slow resistance training have a similar positive effect on AT [9, 13]. However, not all patients seem to respond to the currently used exercise treatments. Silbernagel et al. found at 5 years follow-up that 20% had continued symptoms [14]. In our clinic, we searched for an alternative training approach for those athletes who failed to recover after eccentric training and heavy slow resistance training.

In the treatment of patellar tendinopathy, a training approach based on isometric training has shown interesting perspectives [15]. Inspired by the work and recommendations of Jill Cook, Ebonie Rio, and their groups [16–18], we have developed a treatment program that includes four stages of rehabilitation with the aim of pain reduction, improved function, and returning to sports [19].

The isometric training approach is implemented in all four stages of the protocol and plays a crucial role in the initial stages. Current knowledge regarding AT and isometric rehabilitation is centered on only a few studies [20–23]. Based on exploratory studies and studies assessing pain and stiffness immediately after isometric exercises, there is no evidence that supports an immediate pain relief in AT following isometric exercises [21–23], which is contrary to what has been reported in patellar tendinopathy [24–26]. An AT trial assessing the effect of combining isometric exercises with eccentric training after 3 months found that adding isometric exercises to eccentric training provided no extra benefit [20].

In this small cohort study on chronic AT, our objective was to assess clinical improvement, safety, and feasibility in a home-based 4-stage progressive loading program that included isometric exercises as a key element. The primary outcome was changes in VISA-A score after 12 months. Secondary outcomes were ultrasonographic changes and a change in tenderness on palpation of the tendon after 6 months.

## 2. Methods

### 2.1. Study Design and Participants

A pilot study, designed as a small cohort study with no control group, included 10 patients with chronic midportion AT. The study was planned with respect to the STROBE statement [27].

### 2.2. Inclusion Criteria

- AT symptoms for more than 6 months, with a gradual onset of symptoms in relation to sports activity, causing limitations to exercise.
- A clinical diagnosis of a painful and thickened tendon in relation to activity and on palpation (2 to 7 cm proximal to the insertion on the calcaneus) [6, 28].
- Ultrasonography showing an Achilles tendon with spindle-shaped ultrasonographic thickening of the tendinous tissue of >1 mm in relation to the contralateral tendon and definite signs of tendinopathy (inhomogeneity and hypoechogenicity), with a color Doppler activity of at least grade 2 of 4 (0–4) assessed at baseline [29].

### 2.3. Exclusion Criteria

- Age younger than 18 years.
- Glucocorticoid injections or any other kind of injection into the Achilles tendon within the last 6 months.
- Previous Achilles tendon surgery.
- Insertional AT/enthesopathy.
- Known inflammatory diseases such as rheumatoid arthritis, psoriasis arthritis, or inflammatory bowel disease.
- Ultrasonographic signs of partial ruptures.

The patients were referred to the Department of Sports Medicine at Silkeborg Regional Hospital by general practitioners or other rheumatology/orthopedic departments. Prior to referral, this group of patients had typically been through some kind of rehabilitation, shown in Table 1, managed by their general practitioner and a local physiotherapist. At the first visit to the clinic, either a physician or a physiotherapist would confirm the AT diagnosis with a physical examination and US (ultrasound) examination and define the treatment strategy. If patients were found eligible and wanted to participate in the study, they were given a follow-up appointment with the physician (TPK) responsible for verifying the diagnosis and their inclusion in the study. For those patients who fulfilled the inclusion criteria, the first session with the physiotherapist (TTJ) was booked. In the case of bilateral AT, both tendons were a part of the rehabilitation, but only the more symptomatic tendon was included in the study. The patient decided which tendon should be included for outcome assessment throughout the study, based on symptoms at baseline.

The study protocol was registered with the Danish Data Protection Agency in the Central Denmark Region, (file reference number 1-16-02-856-17). Since the study was based on a treatment already considered standard care, no registration with the local ethics committee was required. The study was carried out in accordance with the principles of the Declaration of Helsinki from the World Medical Association. All enrolled patients provided written informed consent.

### 2.4. Intervention

The rehabilitation protocol was described according to CERT, Consensus on Exercise Reporting Template, recommendations, see Appendix A [30]. The rehabilitation program consisted of four stages, each scheduled for a duration of 1 month. A detailed description of the exercises in each stage was described as suggested by Toigo and Boutellier in Appendix B [31].

Key elements of the program included individualized progressive loading, isometric training, and a limited number of exercises but with many repetitions. The initial part of the program was based on low muscle/tendon loading in order to not induce overload caused by the rehabilitation itself. As tendon and muscle developed strength, adapted to the training, and showed fewer symptoms, the loading was gradually increased on an individual basis in each stage of the program.

All participants started at stage 1. At 1-, 2-, and 3-month follow-ups, instructions for the next stage were given if the individual participant's progress was determined to be satisfactory. If not, based on the participant's symptoms, the physiotherapist determined whether the participant should regress to a lower stage of exercise or simply continue at the same stage for some more weeks before reassessment. Either way, the participant would be scheduled for an additional clinical visit typically within 4–6 weeks, in which the ability to progress to the next stage was reevaluated.

To progress from any given stage to the next, the pain response after loading should not exceed 3 on a numeric rating scale (0–10) and should abate within a few hours. Stage 1 consisted of activating the calf muscles through isometric training, with a focus on pain control. In stage 2, the focus was on increasing the strength in the triceps surae muscle. To progress to stage 3, the participant had to have followed the exercises in stage 2 and participated in activities of daily life with little or no discomfort. In stage 3, there was an increase in training load to accustom the muscles and tendon specifically to the impact of landing (energy storage). Training without impact, jumping or running was allowed. This could be swimming, biking, or using a cross trainer. To progress to stage 4, the participant should have followed the exercises in stage 3 and be able to perform them easily and without discomfort, both during and afterward. In stage 4, in addition to energy storage, the focus was on the push-off and energy release [32]. If running had been discontinued due to the severity of AT, the program recommended waiting until stage 4, where it could be gradually resumed, see Appendix B and Table C. Stage 4 exercises were performed for 1 month. In the following month, participants gradually initiated or increased sporting activities and gradually decreased the frequency of specific stage 4 exercises to a maintenance level of 1–3 times per week. Hereafter, participants were advised to continue life-long/career-long maintenance of calf muscle strength and function by performing stage 2 exercises 1–2 times per week. Besides these specific exercises, the participants were advised to perform supplemental functional training in stages 2, 3, and 4 in terms of walking on toes (forefoot) whenever climbing stairs or walking uphill.

At baseline and follow-up appointments, participants received individual instructions on exercises. Between follow-ups, participants individually performed unsupervised exercises at home.

To increase understanding of, adherence to, and motivation for exercising, participants received an instruction pamphlet with written descriptions and illustrations of exercises as well as advice on which exercises to perform each day of the week, Appendix C. Guidelines for pain management/understanding are stated as follows: “Pain response and intensity are expected to gradually decrease. Exercises should be pain-free or close to pain-free, not exceeding 3 on a 0–10 numeric rating scale. General physical activities, including sports-related activities, can be continued, given no pain response during or after activity; Pain-aggravating activities should be avoided.” Furthermore, participants received verbal information in lay terms on tendon characteristics (including knowledge about how tendons respond to loading), the rationale behind the exercise program, and the goal of the upcoming exercise period.

Exercise instructions were given by a single physiotherapist (TTJ), the developer of this rehabilitation program. TTJ has more than 10 years of experience in treating patients with AT in the Department of Sports Medicine and has in-depth knowledge of the rehabilitation program. As TTJ delivered all instructions and performed all follow-up visits, fidelity to the program was assured.

Throughout the study, patients who did not achieve a satisfying treatment response were offered a glucocorticoid injection (escape strategy).

### 2.5. Outcomes

The baseline characteristics are listed in Table 1. Outcome assessment using the VISA-A questionnaires was performed at visits to the physician and physiotherapist at baseline and at months 1, 2, 3, and 6. A concluding evaluation was carried out by telephone after 12 months. A rheumatologist (TPK) performed an ultrasonographic assessment and clinical examination at baseline and at a 6-month follow-up. The primary outcome was changes in the VISA-A (Victorian Institute of Sports Assessment-Achilles) questionnaire after 12 months, Table 2. VISA-A is a validated and commonly used patient-reported outcome measure, ranging from 0 to 100 points, with 100 indicating optimal function [33, 34]. We used a translated Danish version of the VISA-A [34]. Secondary outcome was tenderness on palpation of the tendon on a 1–10 numeric rating scale when the Achilles tendon was palpated by the physician [29]. The US assessment consisted of changes in tendon thickness, hypoechogenicity, and color Doppler activity as described under “sonographic evaluation” (Table 2). The US examiner was unaware of the patient's current AT status while examining the patient. The patient was informed about the US status only after completion of the VISA-A questionnaire.

Table 3 lists the follow-up questions that addressed the participant's adherence to the rehabilitation program in both the initial stages and the long term. After month 1, 2, and 3, participants were asked if they had followed the rehabilitation on a 5-point Likert scale: 1 = to a very high degree, 2 = to a high degree, 3 = to some degree, 4 = a little, and 5 = not at all. After 12 months they were asked if they were still following the rehabilitation program (yes/no), and, if yes, how many days a week (1–7 days) were they doing the exercises. The questions covered the participants' own feelings of success with the rehabilitation program: how close they felt they were to full recovery from the AT injury (0–100%), to what extent they were able to do what they could before the AT injury (yes/no), whether they had resumed sporting activities (yes/no), and whether they were currently running (yes/no). These secondary outcomes have not been validated. The physiotherapist recorded the following at the 1-, 2-, and 3-month follow-up: the current stage of rehabilitation, if progression to the next stage was recommended, and if the patients should progress further before the next planned control. Decisions regarding whether to change the rehabilitation strategy were also recorded.

Safety and adverse events: All patients were evaluated by US at 6 months for signs of partial rupture of the Achilles tendon.

### 2.6. Sonographic Evaluation

Patients were examined in a prone position with their heels hanging over the examination couch. The ankles were flexed 90 degrees by the examiner, in order to avoid waving of the tendons, for the grayscale examinations of tendon thickness and hypoechogenicity and in relaxed 10 degree plantar flexion for the color Doppler examination to avoid compression of the blood vessels in the tendon [35]. The rheumatologist (TPK) who performed all the US examinations has had more than 15 years of experience with musculoskeletal US. A Hitachi Noblus US scanner (Hitachi Medical) with a 14 MHz linear transducer was used for the clinical examinations. The equipment used to make the US images in this article was a LOGIC E10 (GE Healthcare) with a 15-MHz (MLG-15) linear transducer.

**Figure 1:**
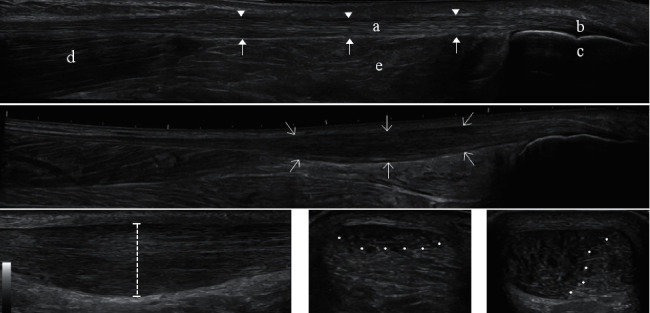
(a) Longitudinal sonogram (extended longitudinal images LOGIQView®) of a healthy Achilles tendon. The length of the shown longitudinal sonogram corresponds to 13 cm. (b) Longitudinal sonogram (extended longitudinal images LOGIQView®) of an Achilles tendon with midportion tendinopathy. (c) Longitudinal sonogram illustrating tendon thickness measurement (dotted line) of a thickened Achilles tendon. (d, e) Transverse sonograms of Achilles tendinopathy illustrating quantification of hypoechogenicity. White dots mark the border between hypoechogenic and normal tendon. The percentage of the entire area that appears hypoechogenic is estimated by eye and corresponds to approximately 25% hypoechogenicity in Figure (d) and 65% in Figure (e). (A) Achilles tendon, (B) the insertion, and enthesis at (C) the calcaneus. (D) Distal section of the soleus muscle at the level of the myotendinous junction. (E) Kager's fat pad. Arrow heads mark the superficial border of the Achilles tendon. Closed arrows mark the deep border of the Achilles tendon. Open arrows surround the tendinopathic part of the Achilles tendon.

Tendon thickness: Figure 1(a) shows the US appearance of a healthy asymptomatic Achilles tendon, with key anatomical structures listed. Figure 1(b) shows an example of a thickened midportion of a tendinopathic Achilles tendon. Figure 1(c) demonstrates how the tendon thickness was measured. The thickest point of the tendons was found in a longitudinal scan and was measured perpendicular to the greatest width of the tendon, the “true” tendon thickness [35, 36]. The thickness was based on the average of three measurements.

Hypoechogenicity: The assessment was defined as a percentage of the tendon that had a hypoechoic appearance of 0–100%, in a transverse scan at the level where the tendon was thickest. A simple “naked eye” assessment was applied. Figures 1(d) and 1(e) show examples of different percentages of hypoechogenicity [37–39].

**Figure 2:**
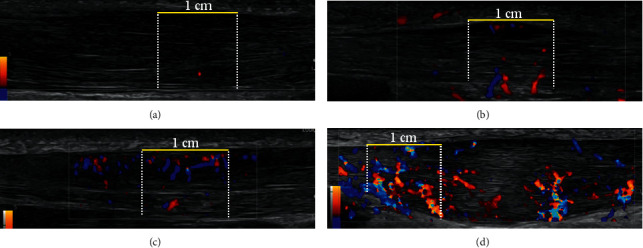
Longitudinal sonogram of Achilles tendinopathy illustrating grading of color Doppler activity from grades 0 to 4. The grading is performed in the region of interest (ROI) defined as a 1 cm longitudinal part of the tendon with maximum color Doppler activity. A horizontal yellow line measuring 1 cm marks the superficial border of the ROI (the superficial border of the Achilles tendon), and white dotted lines mark the proximal and distal borders. The deep border of the ROI is the profound border of the Achilles tendon. Grade 0: no activity (not shown). (a) Grade 1: 1–2 single vessels in the ROI. (b) Grade 2: Doppler activity in less than 25% of the ROI. (c) Grade 3: Doppler activity in 25–50% of the ROI. (d) Grade 4: Doppler activity in more than 50% of the ROI.

Color Doppler activity: The Achilles tendon was examined with color Doppler ultrasonography in the longitudinal plane by moving the transducer from side to side, locating the part with the most Doppler activity. The region of interest (ROI) was in longitudinal scan a 1 cm area limited by the superficial and the profound border of the Achilles tendon at the place with the most Doppler activity. Doppler settings were the same for all patients, with a gain setting just below the noise level and the V-Scale set to 350. We ranked the color Doppler activity from grade 0–4, Figure 2. Grade 0: no activity, grade 1: 1–2 single vessels, grade 2: Doppler activity in less than 25% of the ROI, grade 3: Doppler activity in 25–50% of the ROI, and grade 4: Doppler activity in more than 50% of the ROI [40, 41]. This technique is similar to the modified Öhberg score in which number of vessels is counted on a 0–4 scale [29, 42–48].

### 2.7. Statistical Analysis

Descriptive data are presented as mean ± standard deviation for the continuous data, and in addition, the median and range are presented for the disease duration. Categorical data are presented as numbers and percentages.

The differences from baseline to the various time points were calculated based on paired samples *t*-test. Differences from baselines were changes from baseline to all follow-up time points. The analyses were performed by using SPSS version 17.0 (SPSS Inc., Chicago, IL, USA).

## 3. Results

**Figure 3:**
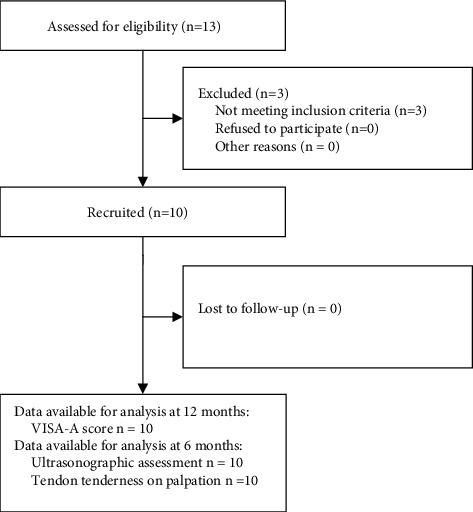
Flow diagram of patients through the study.

Between April 2018 and April 2019, 10 patients were included in the trial, see Figure 3. The baseline characteristics are listed in Table 1. All patients eligible for study inclusion agreed to participate. During the study, no patients were lost to follow-up. All patients were assessed regarding the primary outcome (VISA-A) after 12 months and the ultrasonographic outcomes at 6 months. The post-12-month data by a telephone interview were acquired on average 483 days (range 386 to 615) after study inclusion.

### 3.1. Missing Data

One patient did not show up for the 3-month follow-up. Here, the LOCF (last observation carried forward) was applied.

### 3.2. Clinical Outcomes

**Figure 4:**
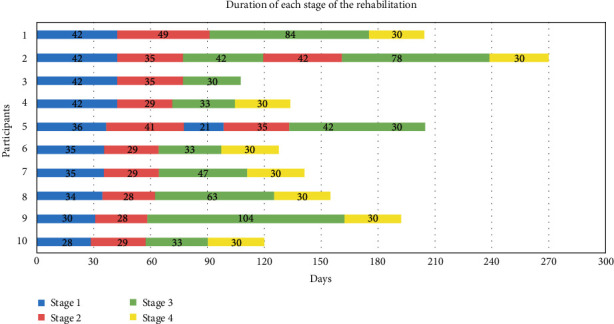
Presentation of the time (days) spent in each of the stages of the rehabilitation program for each participant. Participant number 2 was treated with a glucocorticoid injection 3.5 months into the study. Participant number 3 missed the 3 months follow-up and was therefore not instructed in stage 4 exercises. This participant remained in stage 3 until running was resumed. Participant number 5 was not ready to go to stage 4 at the final follow-up and remained in stage 3.

The primary and secondary outcomes are listed in Table 2. At 12 months, seven participants achieved a VISA-A score of more than 90 points and four participants achieved a score of more than 95 points. Table 3 lists follow-up questions related to the participants' compliance with the rehabilitation program and their return to sports after 12 months.  Figure 4 shows the number of days each participant spent in each stage of the rehabilitation program before being able to progress to the next stage. There was one case of treatment failure in which a glucocorticoid injection was administered after 3.5 months. The injection resulted in a short-term improvement; the VISA-A improved from 45 to 78 at 6-month follow-up. At this point, the patient was instructed in stage 4 exercises. However, the participant's AT relapsed and demonstrated the worst end-of-study outcome of all at 12 months, with a VISA-A score of 57. This participant was not excluded from the study but included in the intention-to-treat population.

Safety: The 6-month US follow-up did not identify any patients with signs suggesting development of partial ruptures.

## 4. Discussion

The available knowledge regarding the clinical effect of isometric rehabilitation is primarily based on studies of patellar tendinopathy [15, 49]. In these patella tendon studies, the isometric tendon loading is thought to have different positive effects. It has a positive mechano-transductive effect on the surrounding aligned fibrillar tissue where the cells convert mechanical stimulus into electrochemical activity, [50] an acute pain reducing effect, [24] and it decreases cortical inhibition [26]. This may allow for the biomechanical tendon profile to normalize [17, 25, 51, 52]. Inspired by the potential effect of isometric training, we developed and implemented a new 4-stage progressive loading program in chronic AT treatment as standard care at the Department of Sports Medicine for patients who had failed to recover after standard rehabilitation. We conducted a cohort study to gain experience and assess the clinical improvement, safety, and feasibility of the treatment prior to setting up a randomized controlled trial (RCT).

The participants included belong to a population that is a priori hard-to-treat, with a mean disease duration of 2.7 years. They had all previously failed to benefit from at least one rehabilitation attempt, and in 90% of the cases, an eccentric training approach had been employed [13].

The baseline characteristics are comparable to those of other published clinical trials on AT, regarding age, gender, disease duration, and sports activity level [13, 29, 53, 54]. At the time of inclusion, the participants had a mean VISA-A score of 52.4. This is similar to what is reported in other intervention studies, with pre-treatment mean VISA-A scores ranging from 24 to 63 points. After 12 months, the mean VISA-A score improved to 87.8 points. Previous studies have shown that healthy individuals have a mean VISA-A score ranging from 96 to 100 points [34]. We observed that of the 10 participants, 7 participants achieved a VISA-A score of more than 90 points and 4 participants achieved a score of more than 95 points. The observed VISA-A score is similar to what Beyer et al. found in an RCT comparing heavy slow resistance training to eccentric training [13]. After 12 months, Beyer et al. found no difference between the groups, with a VISA-A for heavy slow resistance training of 89 points and for eccentric training of 84 points. In a recent systematic review and meta-analysis from 2020 by Clifford et al., only one study on AT and isometric training was found [49]. This study by Gatz et al. investigated whether there was any added benefit from including 1 daily isometric training session to an eccentric exercise program [20]. Both midportion AT and insertional AT were included. After 3 months, both groups improved but without any between-group difference. The mean VISA-A score at baseline was 70.8 and 66.2 in the two treatment arms in the Gatz study compared to 52.4 in our study. The VISA-A improvement after 3 months was similar in the two studies, with a mean improvement of 15 in the Gatz study and 19.1 in our study. The isometric protocol was based on 3 different exercises standing on the toes with increasing load. The exercises had to be performed pain-free. In our protocol the exercises had to be performed 5 times a day with a wide range of isometric exercises. The isometric interventions in the two studies are therefore not directly comparable.

An important aspect of this current rehabilitation protocol is the initial low load approach in order to not overload the tendon and increasing the training load afterward in accordance with symptoms. We aimed at a rehabilitation strategy close to the sports activities that the athletes were trying to return to. Silbernagel et al. documented that athletes can remain in Achilles tendon-loading activities such as running and jumping during rehabilitation [55]. In our study, the participants had stopped running activities prior to joining the study; therefore, the results are not directly comparable. The participants in our study belong to a hard-to-treat subgroup of AT patients for whom eccentric and concentric protocols had failed, which explains why the training approach was different from those used in the studies by Silbernagel, Beyer, and Alfredson [9, 13, 56]. Our program included more attention to pain during and after exercises. The study by Silbernagel et al. used a treatment protocol based on both concentric and eccentric strengthening exercises. The level of acceptable pain during and after physical activity was guided by a pain monitoring model on a 0–10 visual analog scale, allowing 5 as an acceptable score [55,56]. We accepted a pain level of no more than 3 on a numeric rating scale as the exercises had to be performed 5 times a day, and we determined that a more restrictive pain control was required in order to not overload the tendon.

It is generally accepted that tendons need loading in order to recover [57, 58]. We propose that a rehabilitation loading that lies closer to the desired sports activity load could be better than, for instance, protocols in which the rehabilitation is performed either on machines or in anatomical positions far from the anatomical position used in the specific sports activities.

Return-to-sport strategy. Instead of waiting until the rehabilitation program was completed, the participants were allowed, on an individual basis, to resume sports activities gradually during the rehabilitation. In our current study, all participants had stopped running activities due to their AT problems and were not allowed to return to running until stage 4. In milder AT cases, in which the athlete could still be running at some level, we would support athletes continuing to run during the rehabilitation program, but at a level that does not cause tendon overload. We believe that a rehabilitation program which encourages an earlier return to athletic activities will improve the likelihood that participants stay with the program. Despite experiencing failure in a standard course of rehabilitation, participants in this study were able to achieve promising VISA-A results. Furthermore, in this study, the VISA-A score reflected participants who took part in the rehabilitation program as well as a return-to-sport strategy. Ninety percent of the participants in this study had running as their preferred sport before the AT injury. None of the participants was able to run on entering the study. After 12 months, 90% were engaged in sports activities, and 50% were running. This is important since what matters to the athlete is being able to return to sports. As shown in Table 3, it appears that participants closely adhered to the rehabilitation program.

Figure 4 illustrates the amount of time each participant spent in each stage of the program. It shows that the upfront time needed for stages 1 and 2 (1 month each) was generally adequate. In stage 3, the individual adjustment needed was greater, and thus, the variation in time before progression to stage 4 was greater. We did not have an end-date for stage 4 but recommended spending 30 days at this level before increasing sports activities. After having completed the four stages of the rehabilitation program, participants were advised to continue with AT exercises at a maintenance level as described in the Intervention section. We believe that the 4-stage program was easy to follow and that this characteristic is essential to ensure that participants adhere to the planned rehabilitation.

The relative contribution of each of the individual components of this rehabilitation protocol also requires further investigation. Is it the isometric training, the progressive loading strategy, the detailed 4-stage program, the return-to-sport strategy, or the close patient monitoring and guidance from a very experienced physiotherapist? Examining the follow-up data over the 12 months, as shown in Table 2, it appears that the improvement in the VISA-A was evenly distributed over time except that there was no change between 1 and 2 months. Of course, an RCT with a wait-and-see group is needed to determine whether this is simply the result of natural healing over time [59].

The perspectives for a future clinical trial with a proper RCT design are supported by this pilot study. When considering a future RCT at our institution, it appears that recruiting participants within a reasonable time frame would be possible, based on the number of AT patients referred to the clinic. Our experience indicates that the exercises in the program are possible for the participants to perform and the stepwise protocol can be followed, thus assuring that the participants can be retained in the study. Based on patient feedback, the reported 4-stage program was easy to follow and implement in daily life in part because of the short, home-based training sessions without equipment or the need to go to a gym. In addition, we surmise that both the elements of home-training and the early return-to-sport would be attractive to the study participants and favor their completion of the study, thus limiting attrition. A future RCT sample size calculation can be aided by the VISA-A standard deviation (SD) from this pilot study. Compared to the literature, an SD of 24.6, higher than those observed in similar studies where SDs are around 15–20, was achieved most likely due to the small number of participants in our pilot study [13, 29].

Tenderness on palpation is an integral part of the clinical examination in the diagnosis of AT, despite only having modest documentation of the clinical value [6, 60]. We wanted to assess whether tenderness on palpation changed over time in relation to both the VISA-A score and US outcomes. We have previously assessed tenderness on palpation in an RCT on platelet-rich plasma treatment of AT. Here, there was no VISA-A improvement over time or between groups, which was also the case regarding tenderness on palpation [29]. In the current study, tenderness on palpation was reduced by 55% after 6 months, an improvement similar to the VISA-A improvement. Tenderness on palpation could potentially be a prognostic marker. However, tenderness on palpation is not a standardized objective measure such as pressure algometry. We have previously used and documented the value of pressure algometry in AT diagnostics and clinical follow-up, but it is comprehensive and is mainly used in scientific studies [53]. Contrary to pressure algometry, tenderness on palpation is a part of the clinical examination, is an important diagnostic criterion, is easy to perform, and does not require equipment [61, 62]. Consequently, it is worth investigating ways of standardizing this clinical test in future studies. Finally, the reduction in tenderness on palpation corresponded to the reduction in color Doppler activity. A hypothesis could be that a reduced inflammatory response was expressed in both a diminished blood flow and the tenderness on palpation [63, 64].

The ultrasonographic outcomes were assessed at baseline and after 6 months. It is interesting that in our pilot study, some US changes were observed. No statistically significant difference was observed regarding tendon thickness, but the size of the hypoechoic area was significantly reduced, from covering 29.1% of the transverse area at baseline to only 8.5% after 6 months. The degree of heterogeneity (hyper- and/or hypoechogenicity) has been found to be a prognostic marker for poor outcome in a previous study [37]. The degree of color Doppler activity (grade 0–4) was reduced by 50% after 6 months, from a grade 2 to grade 1. In a group of athletes undertaking rehabilitation and gradually attempting to return to sports, it is worth noting that the color Doppler activity decreased while the tendon load increased. Despite the many uncertainties regarding the role of color Doppler activity as a monitoring tool, a reduction in Doppler activity while the symptoms improve is what we theoretically aim for. Again, these data should be addressed with caution since there was no control group.

### 4.1. Limitations

Being a small cohort study with no control group, the study cannot document the effectiveness of the intervention, and no strong conclusions can be drawn from the results. Quantification of the percentage of hypoechogenicity applied in this study in US examination has not been validated or documented. Previous studies simply state whether hypoechogenicity is present or not [37–39]. It is possible that the size of the hypoechoic area could be of importance, which is why we introduce this method for quantification with the aim of obtaining further evidence in future research.

Tenderness on palpation is traditionally used as a present or absent assessment and not scientifically documented when applied to a 0–10 numeric rating scale. Here, a standardized objective measure such as pressure algometry would have improved the reliability of the assessment.

The 12-months' data were based on telephone interviews that, in a few cases, were delayed because the primary investigator was out of the office for a long period of time and the average follow-up time, therefore, ranged from 386 days to 615 days.

## 5. Conclusion and Perspectives

Following a home-based 4-stage rehabilitation program for AT based on a progressive loading strategy including isometric training, we observed an improvement in both VISA-A score and ultrasonographic findings. The study was feasible in terms of a high adherence to the program and with no observed safety issues. The interesting perspectives from this cohort study support a continuous investigation of this specific rehabilitation approach in a future RCT setup.

## Figures and Tables

**Table 1:** Baseline characteristics.

Characteristic	*n* = 10
Age, y	43.1 ± 5.2
Female sex, *n* (%)	1 (10%)
Body mass index, kg/m^2^	27.2 ± 2.7
Height, m	1.84 ± 0.07
Weight, kg	92.5 ± 9.3
Dominant foot, right side, *n* (%)	10 (100%)
Right Achilles tendon affected, *n* (%)	5 (50%)
Bilateral Achilles tendinopathy, *n* (%)	4 (40%)
Duration of symptoms, yr.	2.7 ± 2.7
Median (range)	1.3 (0.5–7.7)
Initial VISA-A score	52.4 ± 24.6
Previous treatments	
Glucocorticoid injection, *n* (%)	2 (20%)
NSAID analgesic use, *n* (%)	3 (30%)
Eccentric exercises, *n* (%)	9 (90%)
Heavy slow resistance training, n (%)	2 (20%)
Did you follow the exercise training plan, yes? *n* (%)	9 (90%)
Preinjury sports	
Running, *n* (%)	9 (90%)
Fitness, *n* (%)	1 (10%)
Football, *n* (%)	1 (10%)
Cycling, *n* (%)	1 (10%)
Floorball, *n* (%)	1 (10%)

Values are mean ± standard deviation unless specified otherwise; *n* = number of participants.

**Table 2:** Outcome measurements.

Outcome	Mean	SE	Difference from baseline (95% CI)	*p* value
VISA-A (0–100)				
Baseline	52.4	7.8		
1 month	63.7	5.2	–11.3 (–22.4 to –0.18)	0.047
2 months	63.5	7.0	–11.1 (–27.3 to 5.1)	0.155
3 months	71.5	5.4	–19.1 (–35.0 to –3.2)	0.024
6 months	79.3	5.2	–26.9 (–42.6 to –11.2)	0.004
12 months	87.8	4.9	–35.4 (–58.0 to –12.8)	0.006
Tenderness on palpation of the tendon (1–10)				
Baseline	5.5	0.6		
6 months	2.5	0.6	3.0 (1.8 to 4.2)	< 0.001
Tendon thickness, mm				
Baseline	9.8	0.8		
6 months	9.3	1.1	0.5 (–0.8 to 1.8)	0.415
Hypoechoic area size, %				
Baseline	29.1	6.5		
6 months	8.5	3.3	20.6 (10.8 to 30.4)	0.001
Color Doppler activity (grade 0–4)				
Baseline	2.0	0.4		
6 months	1.0	0.4	1.0 (0.2 to 1.8)	0.023

VISA-A = Victorian Institute of Sports Assessment-Achilles. SE : Standard Error. CI : Confidence Interval. Difference from baseline is calculated as follows: Baseline minus follow-up. Thus, a negative VISA-A difference is synonymous with an improvement in VISA-A score. The opposite was the case for tenderness on palpation of the tendon and the ultrasonographic outcomes assessed at 6 months; here, there was a positive difference from baseline synonymous with an improvement.

**Table 3:** Follow-up questions at 3 and 12 months.

Question	*n* = 10
Did you follow the rehabilitation program the last month	
After the first month (1–5)	1.8 ± 0.5
After the second month (1–5)	2.3 ± 1.2
After the third month (1–5)	2.4 ± 0.7
Follow-up questions after 12 months	
Are you currently running, yes? *n* (%)	5 (50%)
Do you participate in sports, yes? *n* (%)	9 (90%)
Are you able to do the same as before the Achilles injury, yes? *n* (%)	4 (40%)
If 100% is absolutely perfect, how do you evaluate your Achilles tendon, %	71.3%
Do you still do your Achilles rehabilitation program, yes, *n* (%)	5 (50%)
If you still do your rehabilitation (*n* = 5), how many days a week do you do your exercises, days/week ± SD	3.3 (1.0)

Values are mean ± standard deviation unless specified otherwise; *n* = number of participants. After months 1, 2, and 3 participants were asked if they followed the rehabilitation on a 5-point Likert scale (1–5); 1 = to a very high degree, 2 = to a high degree, 3 = to some degree, 4 = a little, and 5 = not at all.

## Data Availability

The data that support the findings of this study are available from the corresponding author upon reasonable request.

## Supplementary Materials

Supplementary Materials
*Appendix A.* CERT recommendations (Consensus on Exercise Reporting Template). *Appendix B.* Exercise descriptions, as suggested by Toigo and Boutellier, and running retraining. *Appendix C.* Patient handout pamphlet with written and illustrated description of exercises in stages 1–4.

## References

[1] Asplund C. A., Best T. M. (2013). Achilles tendon disorders. *BMJ*.

[2] Magnan B., Bondi M., Pierantoni S., Samaila E. (2014). The pathogenesis of Achilles tendinopathy: a systematic review. *Foot and Ankle Surgery*.

[3] de Jonge S., van den Berg C., de Vos R. J. (2011). Incidence of midportion Achilles tendinopathy in the general population. *British Journal of Sports Medicine*.

[4] Lysholm J., Wiklander J. (1987). Injuries in runners. *The American Journal of Sports Medicine*.

[5] Scott A., Squier K., Alfredson H. (2020). ICON 2019: International scientific tendinopathy symposium consensus: clinical terminology. *British Journal of Sports Medicine*.

[6] Cook J. L., Khan K. M., Purdam C. (2002). Achilles tendinopathy. *Manual Theraphy*.

[7] Fahlstrom M., Jonsson P., Lorentzon R., Alfredson H. (2003). Chronic Achilles tendon pain treated with eccentric calf-muscle training. *Knee Surgery, Sports Traumatology, Arthroscopy*.

[8] Kingma J. J., de Knikker R., Wittink H. M., Takken T. (2007). Eccentric overload training in patients with chronic Achilles tendinopathy: a systematic review. *British Journal of Sports Medicine*.

[9] Alfredson H., Pietila T., Jonsson P., Lorentzon R. (1998). Heavy-load eccentric calf muscle training for the treatment of chronic achilles tendinosis. *The American Journal of Sports Medicine*.

[10] Rowe V., Hemmings S., Barton C., Malliaras P., Maffulli N., Morrissey D. (2012). Conservative management of midportion achilles tendinopathy: a mixed methods study, integrating systematic review and clinical reasoning. *Sports Medicine*.

[11] Habets B., van Cingel R. E. (2015). Eccentric exercise training in chronic mid-portion achilles tendinopathy: a systematic review on different protocols. *Scandinavian Journal of Medicine & Science in Sports*.

[12] van der Vlist A. C., Winters M., Weir A. (2021). Which treatment is most effective for patients with Achilles tendinopathy? A living systematic review with network meta-analysis of 29 randomised controlled trials. *British Journal of Sports Medicine*.

[13] Beyer R., Kongsgaard M., Hougs Kjær B., Øhlenschlæger T., Kjær M., Magnusson S. P. (2015). Heavy slow resistance versus eccentric training as treatment for achilles tendinopathy: a randomized controlled trial. *The American Journal of Sports Medicine*.

[14] Silbernagel K. G., Brorsson A., Lundberg M. (2011). The majority of patients with Achilles tendinopathy recover fully when treated with exercise alone: a 5-year follow-up. *The American Journal of Sports Medicine*.

[15] van Ark M., Cook J. L., Docking S. I. (2016). Do isometric and isotonic exercise programs reduce pain in athletes with patellar tendinopathy in-season? A randomised clinical trial. *Journal of Science and Medicine in Sport*.

[16] Cook J. L., Purdam C. R. (2009). Is tendon pathology a continuum? A pathology model to explain the clinical presentation of load-induced tendinopathy. *British Journal of Sports Medicine*.

[17] Cook J. L., Rio E., Purdam C. R., Docking S. I. (2016). Revisiting the continuum model of tendon pathology: what is its merit in clinical practice and research?. *British Journal of Sports Medicine*.

[18] Rio E., Moseley L., Purdam C. (2014). The pain of tendinopathy: physiological or pathophysiological?. *Sports Medicine*.

[19] Jensen T. T. (2017). Behandling af Achilles tendinopati - en anden vej?. *Dansk Sportsmedicin*.

[20] Gatz M., Betsch M., Dirrichs T. (2020). Eccentric and isometric exercises in achilles tendinopathy evaluated by the VISA-A score and shear wave elastography. *Sports Health*.

[21] Mantovani L., Maestroni L., Bettariga F., Gobbo M., Lopomo N. F., McLean S. (2020). Does isometric exercise improve leg stiffness and hop pain in subjects with Achilles tendinopathy? A feasibility study. *Physical Therapy in Sport*.

[22] O’Neill S., Radia J., Bird K. (2019). Acute sensory and motor response to 45-s heavy isometric holds for the plantar flexors in patients with Achilles tendinopathy. *Knee Surgery, Sports Traumatology, Arthroscopy*.

[23] van der Vlist A. C., van Veldhoven P. L. J., van Oosterom R. F., Verhaar J. A. N., de Vos R. J. (2020). Isometric exercises do not provide immediate pain relief in Achilles tendinopathy: a quasi-randomized clinical trial. *Scandinavian Journal of Medicine & Science in Sports*.

[24] Rio E., Kidgell D., Purdam C. (2015). Isometric exercise induces analgesia and reduces inhibition in patellar tendinopathy. *British Journal of Sports Medicine*.

[25] Rio E., Kidgell D., Moseley G. L., Cook J. (2016). Elevated corticospinal excitability in patellar tendinopathy compared with other anterior knee pain or no pain. *Scandinavian Journal of Medicine & Science in Sports*.

[26] Rio E., van Ark M., Docking S. (2017). Isometric contractions are more analgesic than isotonic contractions for patellar tendon pain: an in-season randomized clinical trial. *Clinical Journal of Sport Medicine*.

[27] Vandenbroucke J. P., von Elm E., Altman D. G. (2007). Strengthening the reporting of observational studies in epidemiology (STROBE): explanation and elaboration. *Annals of Internal Medicine*.

[28] Maffulli N., Kenward M. G., Testa V., Capasso G., Regine R., King J. B. (2003). Clinical diagnosis of Achilles tendinopathy with tendinosis. *Clinical Journal of Sport Medicine*.

[29] Krogh T. P., Ellingsen T., Christensen R., Jensen P., Fredberg U. (2016). Ultrasound-guided injection therapy of Achilles tendinopathy with platelet-rich plasma or saline: a randomized, blinded, placebo-controlled trial. *The American Journal of Sports Medicine*.

[30] Slade S. C., Dionne C. E., Underwood M., Buchbinder R. (2016). Consensus on exercise reporting template (CERT): explanation and elaboration statement. *British Journal of Sports Medicine*.

[31] Toigo M., Boutellier U. (2006). New fundamental resistance exercise determinants of molecular and cellular muscle adaptations. *European Journal of Applied Physiology*.

[32] Cook J. L., Silbernagel K., Griffin S., Alfredson H., Karlson J., Khan K. M., Brukner P., Clarsen B., Cook J. L. (2017). Pain in the achilles region. *Clinical Sports Medicine*.

[33] Robinson J. M., Cook J. L., Purdam C. (2001). The VISA-A questionnaire: a valid and reliable index of the clinical severity of Achilles tendinopathy. *British Journal of Sports Medicine*.

[34] Iversen J. V., Bartels E. M., Langberg H. (2012). The victorian institute of sports assessment - achilles questionnaire (visa-a) - a reliable tool for measuring achilles tendinopathy. *International Journal of Sports Physical Theraphy*.

[35] Fredberg U., Bolvig L., Andersen N. T., Stengaard-Pedersen K. (2008). Ultrasonography in evaluation of Achilles and patella tendon thickness. *Ultraschall in der Medizin*.

[36] Bolvig L., Fredberg U., Rasmussen O. S. (2011). *Textbook On Musculoskeletal Ultrasound - for Beginners and Trained*.

[37] Bakkegaard M., Johannsen F. E., Hojgaard B., Langberg H. (2015). Ultrasonography as a prognostic and objective parameter in Achilles tendinopathy: a prospective observational study. *European Journal of Radiology*.

[38] Cassel M., Baur H., Hirschmuller A., Carlsohn A., Frohlich K., Mayer F. (2015). Prevalence of Achilles and patellar tendinopathy and their association to intratendinous changes in adolescent athletes. *Scandinavian Journal of Medicine & Science in Sports*.

[39] Splittgerber L. E., Ihm J. M. (2019). Significance of asymptomatic tendon pathology in athletes. *Current Sports Medicine Reports*.

[40] Krogh T. P., Fredberg U., Christensen R., Stengaard-Pedersen K., Ellingsen T. (2013). Ultrasonographic assessment of tendon thickness, Doppler activity and bony spurs of the elbow in patients with lateral epicondylitis and healthy subjects: a reliability and agreement study. *Ultraschall in der Medizin - European Journal of Ultrasound*.

[41] Sunding K., Fahlström M., Werner S., Forssblad M., Willberg L. (2016). Evaluation of Achilles and patellar tendinopathy with greyscale ultrasound and colour Doppler: using a four-grade scale. *Knee Surgery, Sports Traumatology, Arthroscopy*.

[42] de Vos R. J., Weir A., Tol J. L., Verhaar J. A. N., Weinans H., van Schie H. T. (2011). No effects of PRP on ultrasonographic tendon structure and neovascularisation in chronic midportion Achilles tendinopathy. *British Journal of Sports Medicine*.

[43] Sengkerij P. M., de Vos R. J., Weir A., van Weelde B. J., Tol J. L. (2009). Interobserver reliability of neovascularization score using power Doppler ultrasonography in midportion achilles tendinopathy. *The American Journal of Sports Medicine*.

[44] Allen G. M., Wilson D. J. (2007). Ultrasound in sports medicine - a critical evaluation. *European Journal of Radiology*.

[45] Boesen A. P., Boesen M. I., Torp-Pedersen S. (2012). Associations between abnormal ultrasound color doppler measures and tendon pain symptoms in badminton players during a season: a prospective cohort study. *The American Journal of Sports Medicine*.

[46] Hirschmuller A., Frey V., Deibert P. (2010). Achilles tendon power Doppler sonography in 953 long distance runners - a cross sectional study. *Ultraschall in der Medizin*.

[47] Hirschmuller A., Konstantinidis L., Frey V. (2011). Prognostic value of achilles tendon doppler sonography in asymptomatic runners. *Medicine & Science in Sports & Exercise*.

[48] O’Connor P. J., Grainger A. J., Morgan S. R., Smith K. L., Waterton J. C., Nash A. F. P. (2004). Ultrasound assessment of tendons in asymptomatic volunteers: a study of reproducibility. *European Radiology*.

[49] Clifford C., Challoumas D., Paul L., Syme G., Millar N. L. (2020). Effectiveness of isometric exercise in the management of tendinopathy: a systematic review and meta-analysis of randomised trials. *BMJ Open Sport and Exercise Medicine*.

[50] Pietrosimone L. S., Blackburn J. T., Wikstrom E. A. (2020). Landing biomechanics are not immediately altered by a single-dose patellar tendon isometric exercise protocol in male athletes with patellar tendinopathy: a single-blinded randomized cross-over trial. *Physical Therapy in Sport*.

[51] Docking S. I., Cook J. (2016). Pathological tendons maintain sufficient aligned fibrillar structure on ultrasound tissue characterization (UTC). *Scandinavian Journal of Medicine & Science in Sports*.

[52] Rio E., Kidgell D., Moseley G. L. (2016). Tendon neuroplastic training: changing the way we think about tendon rehabilitation: a narrative review. *British Journal of Sports Medicine*.

[53] Fredberg U., Bolvig L., Pfeiffer-Jensen M., Clemmensen D., Jakobsen B. W., Stengaard-Pedersen K. (2004). Ultrasonography as a tool for diagnosis, guidance of local steroid injection and, together with pressure algometry, monitoring of the treatment of athletes with chronic jumper’s knee and Achilles tendinitis: a randomized, double-blind, placebo-controlled study. *Scandinavian Journal of Rheumatology*.

[54] de Vos R. J., Weir A., van Schie H. T. (2010). Platelet-rich plasma injection for chronic Achilles tendinopathy: a randomized controlled trial. *JAMA*.

[55] Silbernagel K. G., Thomee R., Eriksson B. I., Karlsson J. (2007). Continued sports activity, using a pain-monitoring model, during rehabilitation in patients with Achilles tendinopathy: a randomized controlled study. *The American Journal of Sports Medicine*.

[56] Grävare Silbernagel K., Thomee R., Thomee P., Karlsson J. (2001). Eccentric overload training for patients with chronic Achilles tendon pain--a randomised controlled study with reliability testing of the evaluation methods. *Scandinavian Journal of Medicine & Science in Sports*.

[57] Heinemeier K. M., Schjerling P., Heinemeier J., Magnusson S. P., Kjaer M. (2013). Lack of tissue renewal in human adult Achilles tendon is revealed by nuclear bomb (14)C. *FASEB Journal*.

[58] Spiesz E. M., Thorpe C. T., Chaudhry S. (2015). Tendon extracellular matrix damage, degradation and inflammation in response to in vitro overload exercise. *Journal of Orthopaedic Research*.

[59] Smidt N., Van der Windt D. A., Assendelft W. J., Deville W. L., Korthals-de Bos I. B., Bouter L. M. (2002). Corticosteroid injections, physiotherapy, or a wait-and-see policy for lateral epicondylitis: a randomised controlled trial. *The Lancet*.

[60] Matthews W., Ellis R., Furness J., Hing W. A. (2021). The clinical diagnosis of Achilles tendinopathy: a scoping review. *PeerJ*.

[61] Reiman M., Burgi C., Strube E. (2014). The utility of clinical measures for the diagnosis of achilles tendon injuries: a systematic review with meta-analysis. *Journal of Athletic Training*.

[62] Hutchison A. M., Evans R., Bodger O. (2013). What is the best clinical test for Achilles tendinopathy?. *Foot and Ankle Surgery*.

[63] Millar N. L., Silbernagel K. G., Thorborg K. (2021). Tendinopathy. *Nature Reviews: Disease Primers*.

[64] Fredberg U., Stengaard-Pedersen K. (2008). Chronic tendinopathy tissue pathology, pain mechanisms, and etiology with a special focus on inflammation. *Scandinavian Journal of Medicine & Science in Sports*.

