# Supplementary material for: An Isometric and Functionally Based 4-Stage Progressive Loading Program in Achilles Tendinopathy: A 12-Month Pilot Study
Source: Transl Sports Med. 2022 May 24;2022:6268590. doi: 10.1155/2022/6268590 (PMC11022783; doi:10.1155/2022/6268590)
Supplement: Supplementary Materials — Appendix A. CERT recommendations (Consensus on Exercise Reporting Template). Appendix B. Exercise descriptions, as suggested by Toigo and Boutellier, and running retraining. Appendix C. Patient handout pamphlet with written and illustrated description of exercises in stages 1–4. [file 6268590.f1.zip › 6268590.f1/Appendix A. CERT.docx]

## Appendix A

## CERT - Consensus on Exercise Reporting Template

## A Checklist for what to include when reporting exercise programs

| Section/Topic | Item # | Checklist item | Location (page, table, appendix) |
| --- | --- | --- | --- |
| WHAT:  materials | 1 | Detailed description of the type of exercise equipment (e.g. weights, exercise equipment such as machines, treadmill, bicycle ergometer etc) | Appendix B |
| WHO:  provider | 2 | Detailed description of the qualifications, teaching/supervising expertise, and/or training undertaken by the exercise instructor | Intervention section |
| HOW:  delivery | 3 | Describe whether exercises are performed individually or in a group | Intervention section |
|  | 4 | Describe whether exercises are supervised or unsupervised and how they are delivered | Intervention section |
|  | 5 | Detailed description of how adherence to exercise is measured and reported | Intervention section |
|  | 6 | Detailed description of motivation strategies | Intervention section |
|  | 7a | Detailed description of the decision rule(s) for determining exercise progression | Intervention section |
|  | 7b | Detailed description of how the exercise program was progressed | Appendix B and Appendix C |
|  | 8 | Detailed description of each exercise to enable replication (e.g. photographs, illustrations, video etc) | Appendix B and Appendix C |
|  | 9 | Detailed description of any home program component (e.g. other exercises, stretching etc) | Intervention section |
|  | 10 | Describe whether there are any non-exercise components (e.g. education, cognitive behavioural therapy, massage etc) | Intervention section |
|  | 11 | Describe the type and number of adverse events that occurred during exercise | Clinical Outcomes section - safety |
| WHERE:  location | 12 | Describe the setting in which the exercises are performed | Methods section |
| WHEN, HOW MUCH:  dosage | 13 | Detailed description of the exercise intervention including, but not limited to, number of exercise repetitions/sets/sessions, session duration, intervention/program duration etc. | Appendix B and Appendix C |
| TAILORING: what, how | 14a | Describe whether the exercises are generic (one size fits all) or tailored to the individual | Intervention section |
|  | 14b | Detailed description of how exercises are tailored to the individual | Intervention section |
|  | 15 | Describe the decision rule for determining the starting level at which people commence an exercise program (such as beginner, intermediate, advanced etc.) | Intervention section |
| HOW WELL: planned, actual | 16a | Describe how adherence or fidelity to the exercise intervention is assessed/measured | Intervention section |
|  | 16b | Describe the extent to which the intervention was delivered as planned | Result and Discussion section |
