# Supplementary material for: An Isometric and Functionally Based 4-Stage Progressive Loading Program in Achilles Tendinopathy: A 12-Month Pilot Study
Source: Transl Sports Med. 2022 May 24;2022:6268590. doi: 10.1155/2022/6268590 (PMC11022783; doi:10.1155/2022/6268590)
Supplement: Supplementary Materials — Appendix A. CERT recommendations (Consensus on Exercise Reporting Template). Appendix B. Exercise descriptions, as suggested by Toigo and Boutellier, and running retraining. Appendix C. Patient handout pamphlet with written and illustrated description of exercises in stages 1–4. [file 6268590.f1.zip › 6268590.f1/Appendix B. Exercise descriptions.docx]

###### Table A. Strengthening exercise descriptors

|  | **Single-leg toe stand** | **Single-leg heel raises** |
| --- | --- | --- |
| **Used in stage** | 1,2,3, & 4 | 2,3, & 4 |
| **Load** | Full body weight | Stage 2: Full body weight  Stage 3: Full body weight + a few kilograms in backpack  Stage 4: Full body weight + 5–10 kilograms in backpack |
| **Repetitions** | 1 | Stages 2 & 3: 6 |
|  |  | Stage 4: 6 with progression to 8 |
| **Set**  **(per session)** | Stage 1: 5 | Stages 2 & 3: 4 |
|  | Stages 2 & 3: 3 |  |
|  | Stage 4: 2 | Stage 4: 4 with progression to 5 |
| **Rest between sets** | 2 min | 2 min |
| **Sessions**  **(per week)** | Stage 1: 28–35  *(4*–*5 sessions each day, 7 days per week)* | Stage 2:   - First 2–3 weeks: 4 - After 2–3 weeks: 7 |
|  | Stage 2:   - First 2–3 weeks: 16   (2–3 times a day, 6 days per week, once the last day)   - After 2–3 weeks: 13   *(twice a day, 6 days per week, once the last day)* |  |
|  | Stage 3: 11  *(twice a day on days 1,3,5, & 7, once a day on days 2,4, & 6)* | Stage 3: 4 |
|  | Stage 4: 3  *( days 2, 4, & 6)* | Stage 4: 3 |
| **Duration of**  **training period** | 4 months (1 month in each stage) | 3 months (1 month in each stage 2,3, & 4) |
| **Contraction mode** | 45 seconds isometric | Alternate concentric and eccentric contraction performed at a moderate pace (about 2 seconds concentric and 2 seconds eccentric) |
| **Rest between repetitions** | Not applicable | 0 seconds |
| **Time under tension**  **seconds/exercise/ session** | Stage 1: 225 (5 x 45) | -  Stage 2: 96 (4x6x4)  Stage 3: 96 (4x6x4)  Stage 4: 96 progressing to 160 (4x6x4 – progressing to 4x8x5) |
|  | Stage 2: 135 (3x45) |  |
|  | Stage 3: 135 (3x45) |  |
|  | Stage 4: 90 (2x45) |  |
| **Contraction failure**  **in each set** | No | No |
| **Range of motion** | No | Maximum possible |
| **Rest between sessions** | Stage 1:  3-4 hours during daytime, about 10 hours from the late-night session until first session the next day | Stage 2:   - First 2–3 weeks:   48 hours between exercise on days 1, 3, 5, & 7 – and 24 hours between exercise on days 7 & 1   - After 2–3 weeks:   24 hours |
|  | Stage 2:   - First 2–3 weeks:   Days 1, 3, & 5: 12 hours, Days 2 (6 hours), 4 (6 hours), & 6 (12 hours). Day 7: 24 hours   - After 2–3 weeks:   Days 1–6: 12 hours, Day 7: 24 hours |  |
|  | Stage 3:  Week days 1,3,5 & 7: 12 hours, Week days 2,4, & 6: 24 hours | Stage 3:  Days 1,3,5, & 7: 48 hours  Between days 7 & 1 (in next week): 24 hours |
|  | Stage 4:  48 hours between days 2, 4, & 6.  72 hours between days 6 & 2 (in the next week) | Stage 4:  48 hours between days 2, 4, & 6.  72 hours between days 6 & 2 (in next week) |
| **Anatomical definition of the exercises** | Upright standing position on single leg (target leg) with support of, e.g., a solid table or a wall. The ankle is fully plantar-flexed to obtain toe-standing position. Pressure is at first and second metatarsophalangeal joints and with the knee straight. | Single-leg (target leg) heel rise in an upright standing position with support of, e.g., a solid table or a wall. In a slow pace the ankle is fully plantar-flexed by contraction of mm. soleus, gastrocnemii, flexor hallucis longus, and flexor digitorum. At toe-standing position, pressure is on first and second metatarsophalangeal joints. Finally, the heel is lowered until full contact with the floor. The knee is kept straight throughout the exercise. |
| **Illustration** | 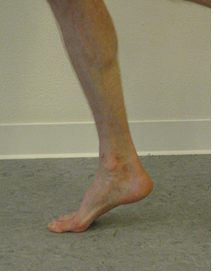 |  |

Table B. Dose-specific functional exercise descriptors

|  | **Toe walking** | **Lunges** | **Backward stride** | **Box-jump** | **Forefoot jump** |
| --- | --- | --- | --- | --- | --- |
| **Used in stage** | 2,3, & 4 | 3 & 4 | 3 & 4 | 4 | 4 |
| **Load** | Full body weight | Body weight | Body weight | Full body weight | Full body weight |
| **Repetitions** | Not applicable | 15 | 15 | 6 | Not applicable (duration 30 seconds) |
| **Rest between repetitions** | Not applicable | Repetitions are performed at a moderate pace without defined rest | Repetitions are performed at a moderate pace without defined rest | Repetitions are performed at a moderate pace without defined rest | 0 seconds |
| **Set (per session)** | 1 | 3 | 3 | 3 | 3 |
| **Rest between sets** | Not applicable | As needed. Not defined. | A needed. Not defined. | As needed. Not defined. | As needed. Not defined. |
| **Sessions (per week)** | 7 | 3 | 3 | 3 | 3 |
| **Duration of training period** | 3 months (1 month in each of stages 2, 3, & 4) | 2 months (1 month in each of stages 3 & 4) | 2 months (1 month in each of stages 3 & 4) | 1 month | 1 month |
| **Rest between sessions** | 24 hours | Between days 1,3,5, & 7: 48 hours  Between days 7 and 1 (in next week): 24 hours | Between days 1,3,5, & 7: 48 hours  Between days 7 and 1 (in next week): 24 hours | Between days 1,3,5, & 7: 48 hours  Between days 7 and 1 (in next week): 24 hours | Between days 1,3,5, & 7: 48 hours  Between days 7 and 1 (in next week): 24 hours |
| **Anatomical definition of the exercises** | Upright walking with knees straight and ankles as close to full plantar-flexion as possible. | Upright standing position on both legs. The target leg slowly steps forward into a forefoot landing. In the landing, the ankle is kept as close to full plantar-flexion as possible. Mm. soleus, gastrocnemii, flexor hallucis longus and flexor digitorum are maximally contracted, working like a tensioned spring without giving in. The foot of the non-target leg stays behind with very little weight bearing, but always more or less in contact with the floor.  In the landing position, tibia of the target leg is positioned right above the ankle and the upper body is kept upright.  Once comfortable in the exercise, the load is increased by taking longer steps and shifting on more weight to the target leg. | Upright standing position on a small, stable step with a height of 15 (12–18) cm. Take a step backwards and down with the target leg. Landing is on the forefoot with the ankle as close to maximal plantar-flexion as possible. Mm. soleus, gastrocnemii, flexor hallucis longus and flexor digitorum are maximally contracted, working like a tensioned spring without giving in. Upper body is kept upright.  Then return to the starting position using the non-target leg.  Once comfortable in the exercise, load is increased by shifting on more weight to the target leg. | Upright standing position facing a small, stable step or box with a height of 15 (12–18) cm. Body weight is evenly distributed on both legs.  First, muscles are pre-tensioned by a small flexion of the knees and a small heel raise.  Second, jump onto the step by extending the knees and plantar-flexing the ankles.  Landing is on the forefeet with the ankle as close to maximal plantar-flexion as possible. Knees are slightly bent. Shortly after, slowly step down to starting position. | Upright standing position on a plane surface.  Raise the heels until ankles are fully plantar-flexed and keep this position with pressure at first and second metatarsophalangeal joints. Knees are kept straight throughout the exercise.  Make small, fast, repetitive, upward jumps/landings while keeping the ankle as close to full plantar-flexion as possible. Mm. soleus, gastrocnemii, flexor hallucis longus, and flexor digitorum are working like a tensioned spring. |
| **Illustration** | 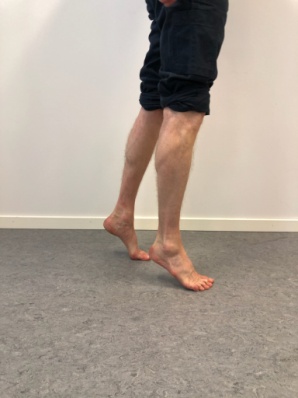 | 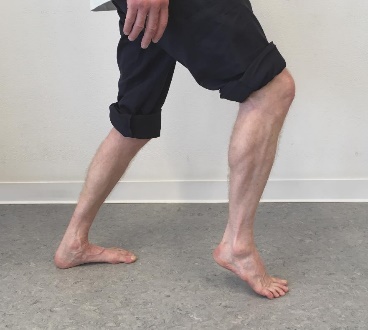 | 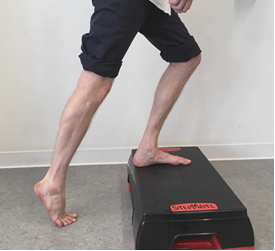 | 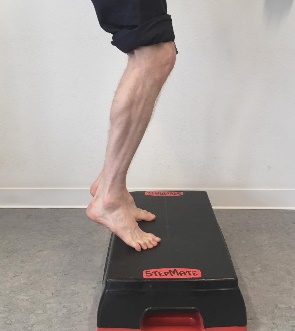 | 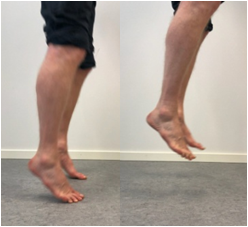 |

**Table C: Running retraining.**

*Running sessions are performed twice a week (days 1 and 5). During the first 4 weeks, sessions contain both running and walking.*

| Guidelines in general | Start by running/walking 1.5 km. Running is at 60% of maximum speed.  Distance is increased by 15% per week until a distance of 4 km is obtained. Hereafter, weekly increase is 10% only. |
| --- | --- |
| Exercises performed during running | Running 100 meters on toes on plain ground. Performed twice per session  Running uphill (stairs or hill). 50 steps 2–3 times per session. During weeks 1 & 2 walk downhill. From week 3 and onward, running down can be included, giving that it can be done without lowering the heel too much (must not touch the ground) after landing. |
| Week 1 | Alternately, 1 minute running and 1 minute walking |
| Week 2 | Alternately, 2 minutes running and 1 minute walking |
| Week 3 | Alternately, 3 minutes running and 1 minute walking |
| Week 4 | Alternately, 4 minutes running and 1 minute walking |
| Week 5 and forward | Running |
