# Supplementary material for: An Isometric and Functionally Based 4-Stage Progressive Loading Program in Achilles Tendinopathy: A 12-Month Pilot Study
Source: Transl Sports Med. 2022 May 24;2022:6268590. doi: 10.1155/2022/6268590 (PMC11022783; doi:10.1155/2022/6268590)
Supplement: Supplementary Materials — Appendix A. CERT recommendations (Consensus on Exercise Reporting Template). Appendix B. Exercise descriptions, as suggested by Toigo and Boutellier, and running retraining. Appendix C. Patient handout pamphlet with written and illustrated description of exercises in stages 1–4. [file 6268590.f1.zip › 6268590.f1/Appendix C. Patient handout pamphlet.docx]

Stage 1


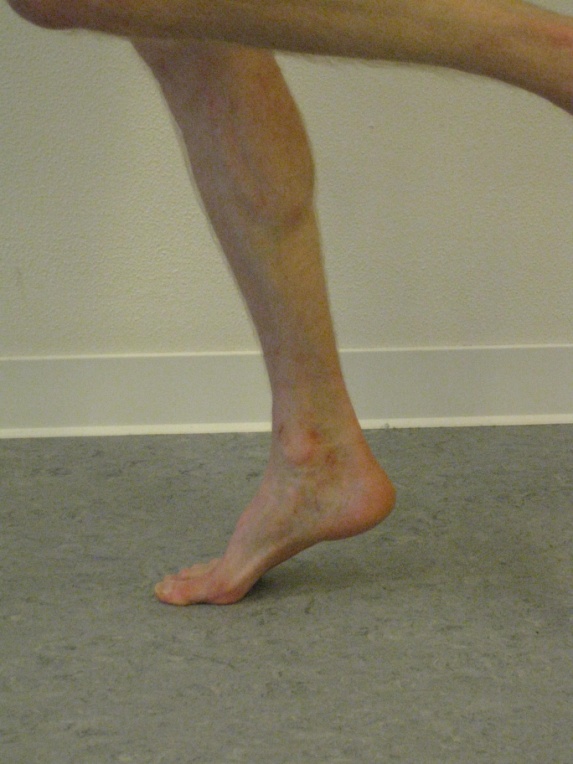


Stand on one leg and support yourself with hand on a wall or a table to prevent balancing movements.

Stand on your toes and hold this position with a pressure on first and second toe, Figure 1. Keep the knee stretched at all times.

Carry out the exercise 4–5 times daily, 5 x 45 sec with a 2-minute pause between each repetition.

Use a watch to make sure that the training dose is correct.

Figure 1. Toe stand

Note:

Use the other foot for light support rather than do a wobbly stand on one leg, Figure 2:

- Lack of heel lift
- Bent knee
- Support on the lateral side of the foot

Reduce the height of the heel lift if pain occurs.

Stage 2

**Functional strength training**

**
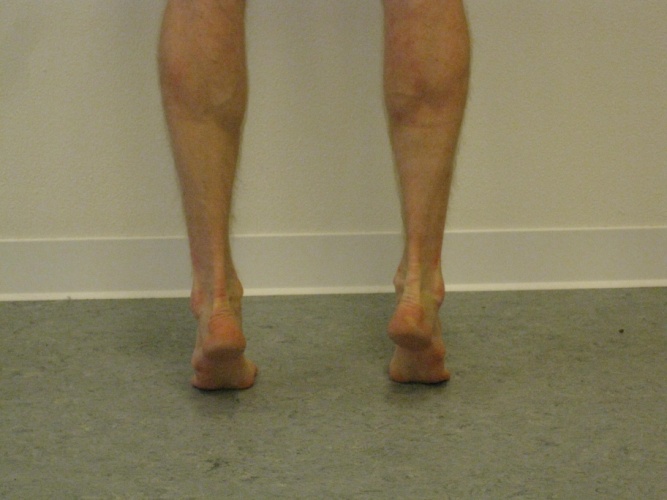
**

Figure 3. Heel raises


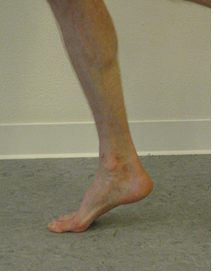


Walk on the very tips of your toes, Figure 2. Heels must not fall! 2 minutes daily.

Toe walking on steps when you climb stairs - only upwards and on the very tips. Heels must not fall.

**Strength training of calf**

T = toe stand (isometric training) on single-leg only.

45 sec. x 3, carried out 2–3 times a day, 7 days a week (schedules A + B)

Figure 2. Toe walking

H = single-leg heel raises (dynamic training), Figure 3.

4 x 6 repetitions with a 2-minute pause between each set, 4 days a week (Schedule A)

X = indicates the functional strength training.

Schedule A: Training program the first 2-3 weeks

| **Outline** | Monday | Tuesday | Wednesday | Thursday | Friday | Saturday | Sunday |
| --- | --- | --- | --- | --- | --- | --- | --- |
| morning | T | T | T | T | T | T | T |
| noon | H | T | H | T | H | T | H |
| evening | T | T | T | T | T | T |  |
| Functional  strength | X | X | X | X | X | X | X |

Increase the dynamic training after 2-3 weeks with single-leg heel lifts so that the exercises are carried out daily. (Schedule B)

Schedule B: Training program after 2-3 weeks

| **Outline** | Monday | Tuesday | Wednesday | Thursday | Friday | Saturday | Sunday |
| --- | --- | --- | --- | --- | --- | --- | --- |
| morning | T | T | T | T | T | T | T |
| noon | H | H | H | H | H | H | H |
| evening | T | T | T | T | T | T |  |
| Functional strength | X | X | X | X | X | X | X |

Stage 3


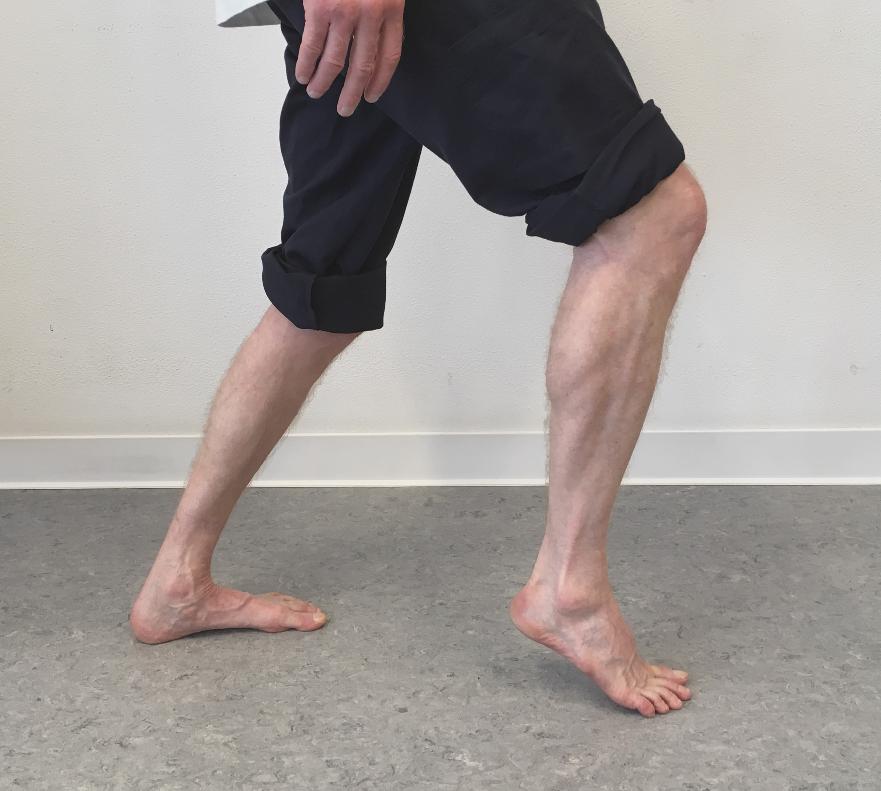


Figure 4. Lunges

**Strength training of calf**

Toe stand (Isometric training) carried out 45 seconds x 3, 1-2 times a day, morning and evening (see schedule).

Heel raises (Dynamic training) 4 x 6 repetitions, 3-4 times a week. Eventually, with a few kilos in a backpack.

**Functional strength training**

Continue toe walking on stairs/hills as in phase 2, **plus** 2 minutes’ toe walking on a level floor.

Lunge forward and land on forefoot, Figure 4. Take a step forward and land high on your forefoot, keeping the heel elevated.

3 x 15 repetitions, 3 times a week.


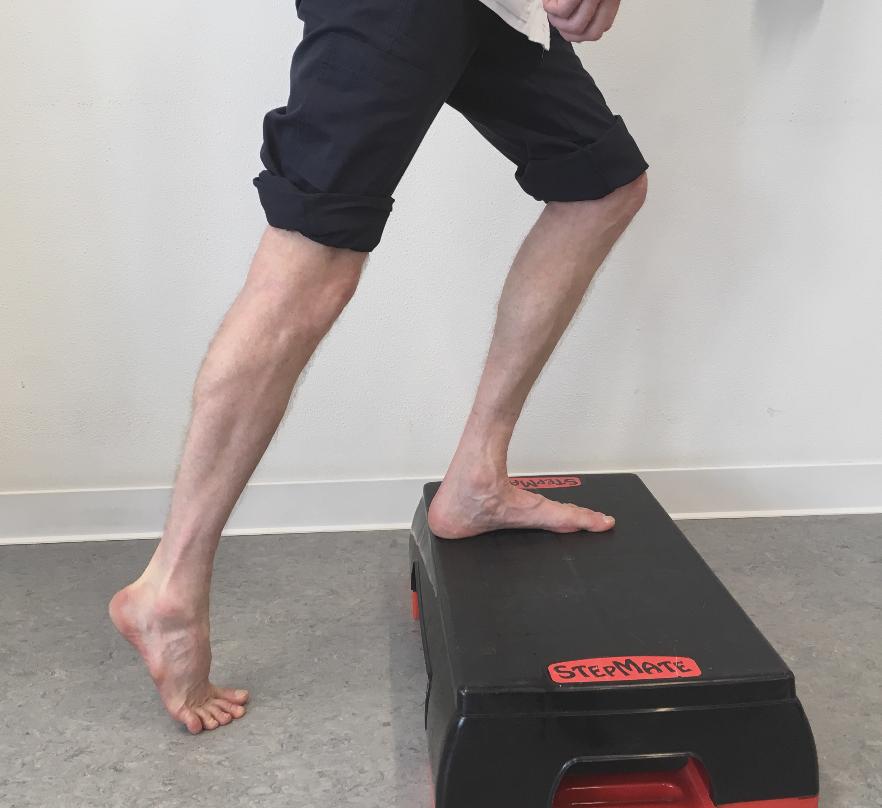


Figure 5. Backward stride

Backward stride. Start on floor.

Progress to starting from standing on step (15 cm high).

Stand on a box, take a step backwards and land high on your forefoot with knee stretched and without letting the heel drop very much, Figure 5.

3 x 15 repetitions, 3 times a week.

Schedule C

| **Outline** | Monday | Tuesday | Wednesday | Thursday | Friday | Saturday | Sunday |
| --- | --- | --- | --- | --- | --- | --- | --- |
| Toe-stand  45 sec. x 3 | morning & evening | morning | morning & evening | morning | morning & evening | morning | morning & evening |
| Functional strength | X | X | X | X | X | X | X |
| Heel-raises  4 x 6 | X |  | X |  | X |  | X |
| Forward lunges 3 x 15 |  | X |  | X |  | X |  |
| Backward strides  3 x 15 |  | X |  | X |  | X |  |

Stage 4


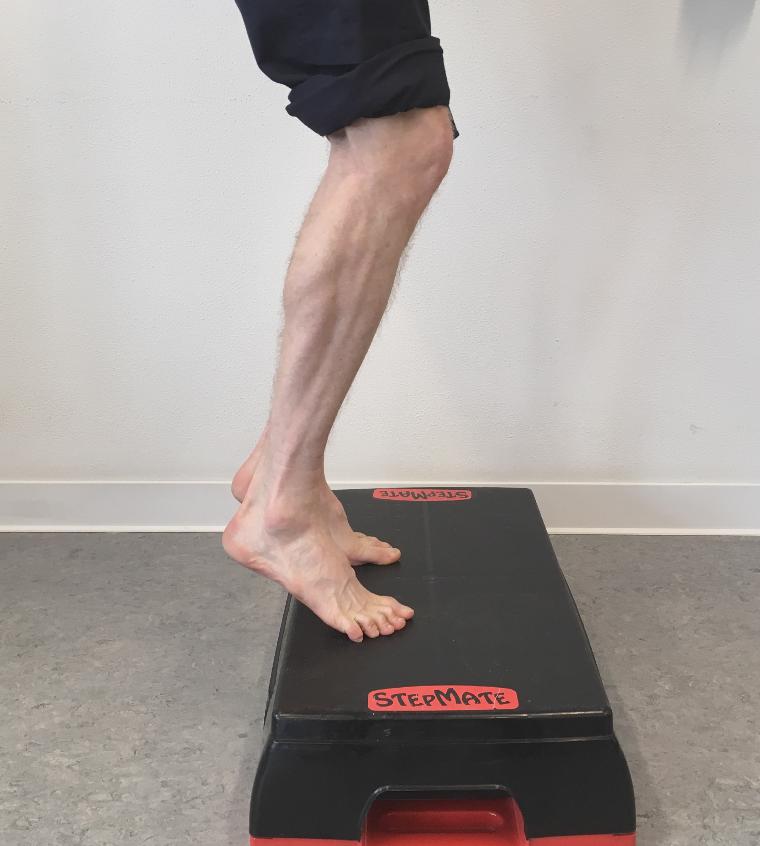


Figure 6. Box-jump

**Strength training of calf – 3 days a week (see schedule D)**

Toe stand 2 x 45 seconds

Heel raises 4 x 6 repetitions – eventually, 5 x 8 repetitions + optionally extra load with 5–10 kilos in a backpack.

**Functional strength training – 3 times a week (see schedule D)**

Toe walking on stairs/hill plus 2-minute toe walking on a level floor.

Backward strides with landing on forefoot, 3 x 15 repetitions.

Lunges forward with landing on forefoot, 3 x 15 repetitions.

Hop on to a box (box jump) landing on forefoot, Figure 6. Begin with 15 cm, 3 x 6 repetitions.

Skipping or jumping on forefoot on the spot. Begin with 30 seconds x 3, and slowly increase with intervals of 10–15 seconds.

**Running twice a week (see schedule D)**

Begin with 1.5 km at 60% of maximum speed. The distance is increased by 15% a week. After reaching 4 km, the distance is only increased by 10% a week. During the first 4 weeks there are walking pauses in between the running intervals.

Week 1: 1 minute running, 1 minute walking.

Week 2: 2 minutes running. 1 minute walking.

Week 3: 3 minutes running, 1 minutes walking.

Week 4: 4 minutes running, 1 minutes walking.

Week 5 and onward: Running without walking intervals.

Exercises during a running session:

- Run on toes/forefoot straight ahead 100 meters a couple of times during your tour.
- Run on toes upward on stairs or hills. 50 steps, 2-3 times during your run.
- Walk to the bottom of the stairs or hill the first 2 weeks. From then on you may run down the stairs if you can without the heel touching the steps.

Schedule D

| **Outline** | Monday | Tuesday | Wednesday | Thursday | Friday | Saturday | Sunday |
| --- | --- | --- | --- | --- | --- | --- | --- |
| Running | X |  |  |  | X |  |  |
| Calf strength |  | X |  | X |  | X |  |
| Functional-strength | X |  | X |  | X |  |  |
